# Supplementary material for: Combined statistical-biophysical modeling links ion channel genes to physiology of cortical neuron types
Source: bioRxiv. 2025 Jan 2:2023.03.02.530774. Preprint. [Version 2] doi: 10.1101/2023.03.02.530774 (PMC11722265; doi:10.1101/2023.03.02.530774)
Supplement: 1 [file NIHPP2023.03.02.530774V2-supplement-1.pdf]

bioRxiv preprint doi: https://doi.org/10.1101/2023.03.02.530774; this version posted January 2, 2025. The copyright holder for this preprint (which was not certified by peer review) is the author/funder, who has granted bioRxiv a license to display the preprint in perpetuity. It is made available under aCC-BY 4.0 International license.

SUPPLEMENTARY MATERIAL

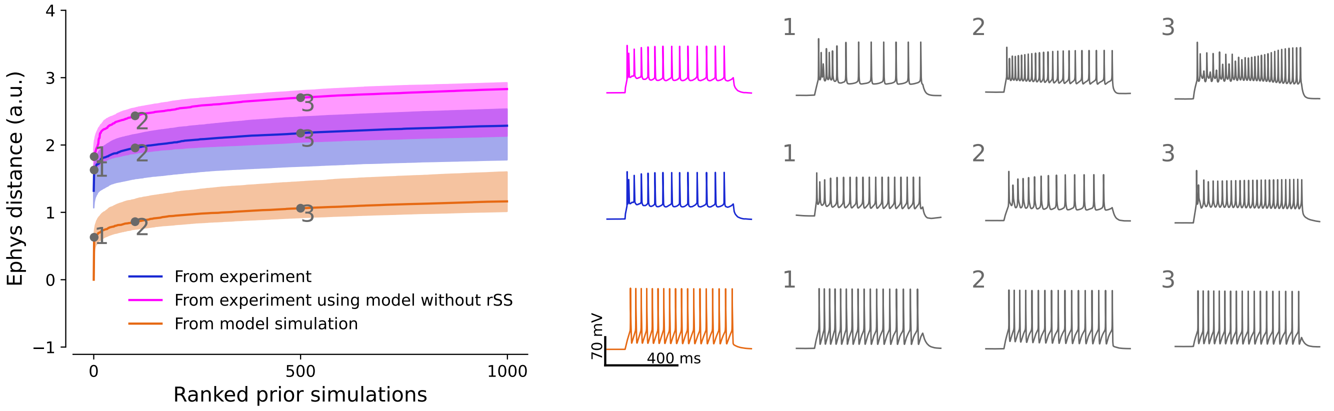

Figure S1 Model misspecification with and without scaling r_{SS} parameter. Analogous to Fig. 4b, but including model simulations without r_{SS} parameter.

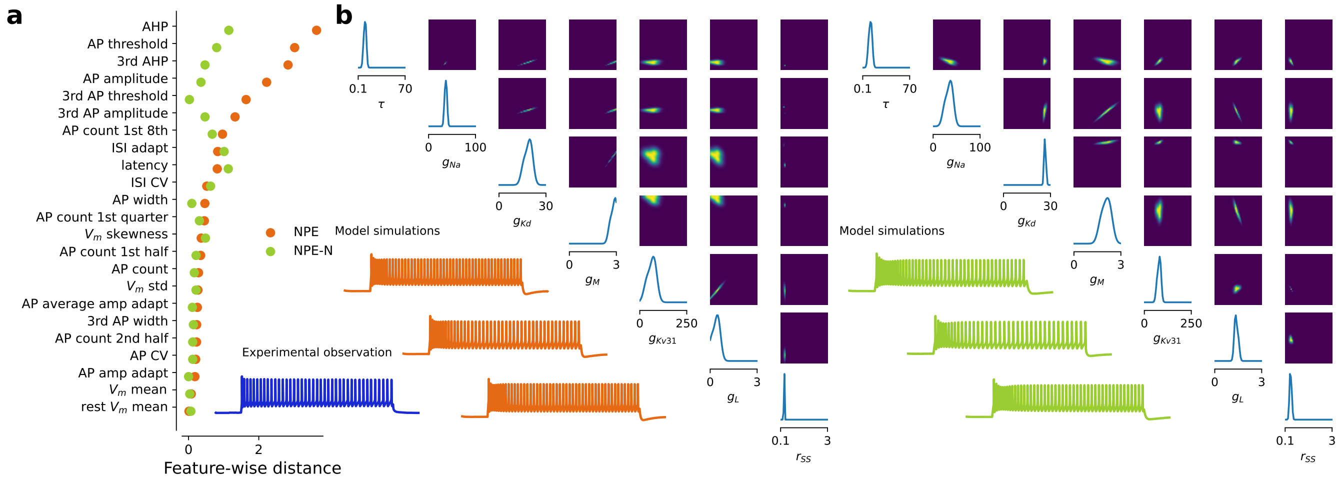

Figure S2 NPE vs NPE-N, illustration 1: fast-spiking *Pvalb Calb1_1* interneuron. Analogous to Fig. 3a,b.

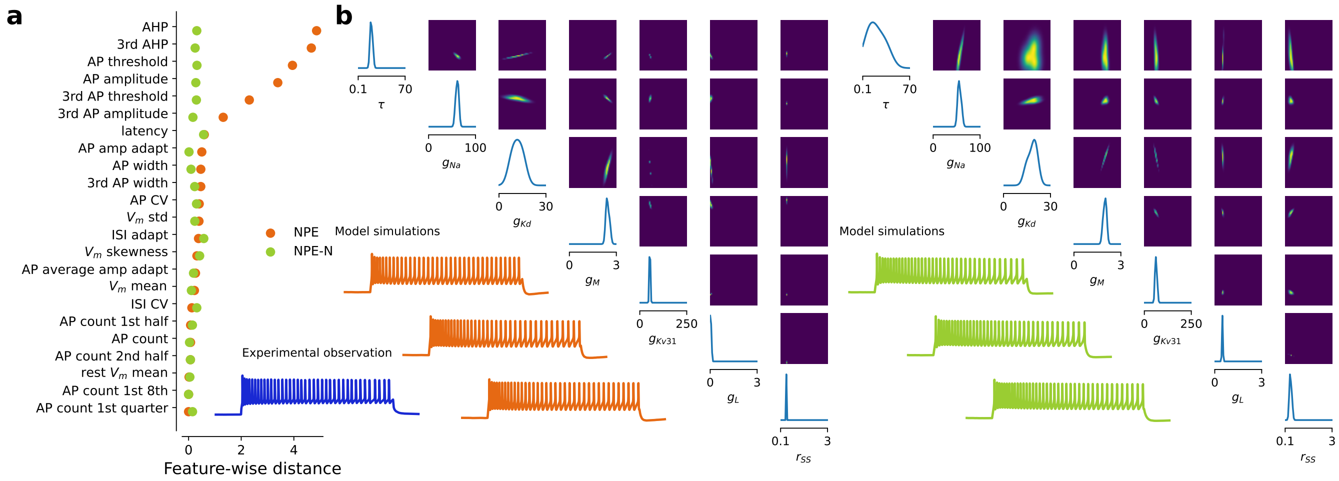

Figure S3 NPE vs NPE-N, illustration 2: *Sst Crhr2_1* interneuron. Analogous to Fig. 3a,b.

bioRxiv preprint doi: https://doi.org/10.1101/2023.03.02.530774; this version posted January 2, 2025. The copyright holder for this preprint (which was not certified by peer review) is the author/funder, who has granted bioRxiv a license to display the preprint in perpetuity. It is made available under aCC-BY 4.0 International license.

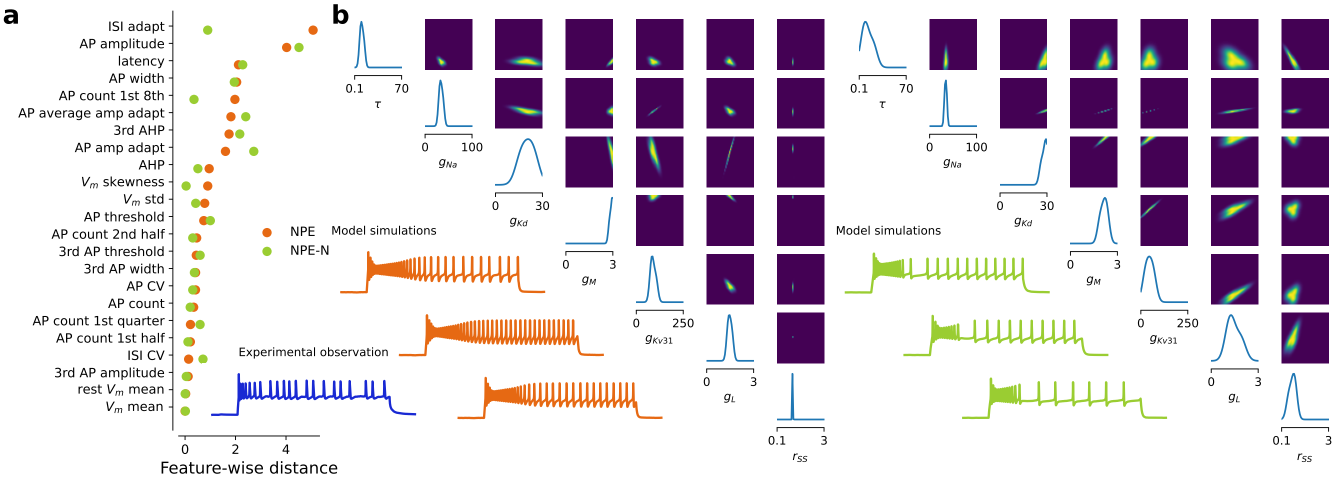

Figure S4 **NPE vs NPE-N, illustration 3: *Vip Serpinf1_1* interneuron.** Analogous to Fig. 3a,b.

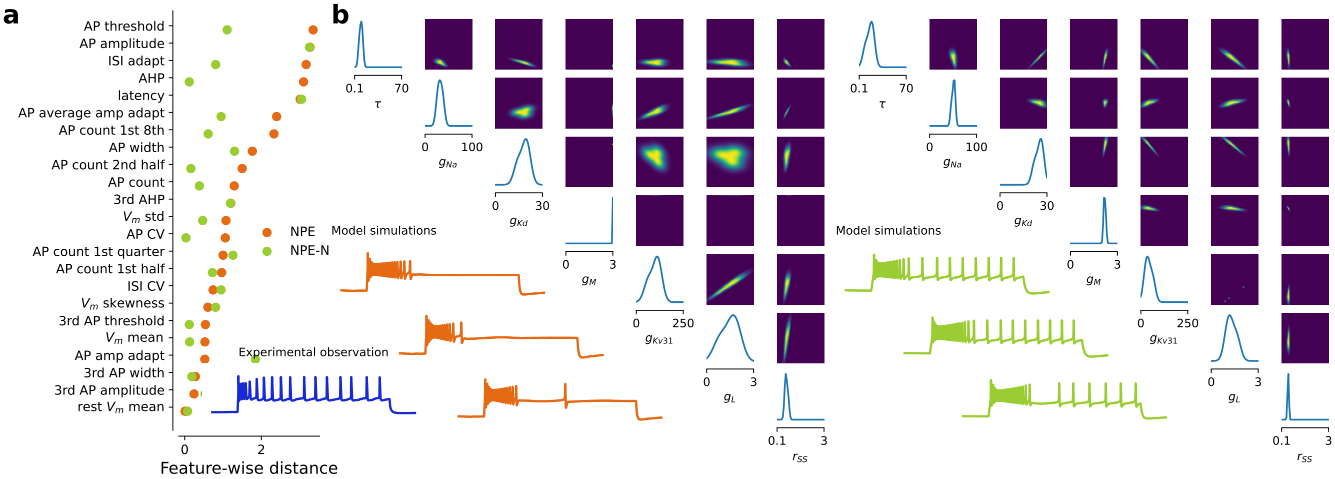

Figure S5 **NPE vs NPE-N, illustration 4: *Lamp5 Egln3_1* interneuron.** Analogous to Fig. 3a,b.

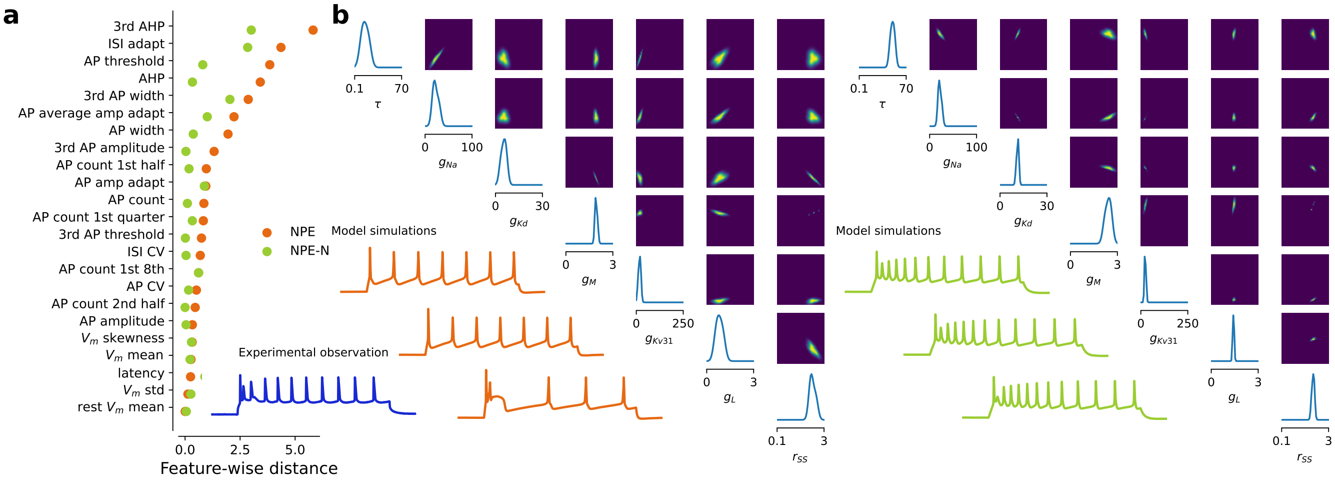

Figure S6 **NPE vs NPE-N, illustration 5: *L6 CT Cpa6* pyramidal cell.** Analogous to Fig. 3a,b.

bioRxiv preprint doi: https://doi.org/10.1101/2023.03.02.530774; this version posted January 2, 2025. The copyright holder for this preprint (which was not certified by peer review) is the author/funder, who has granted bioRxiv a license to display the preprint in perpetuity. It is made available under aCC-BY 4.0 International license.

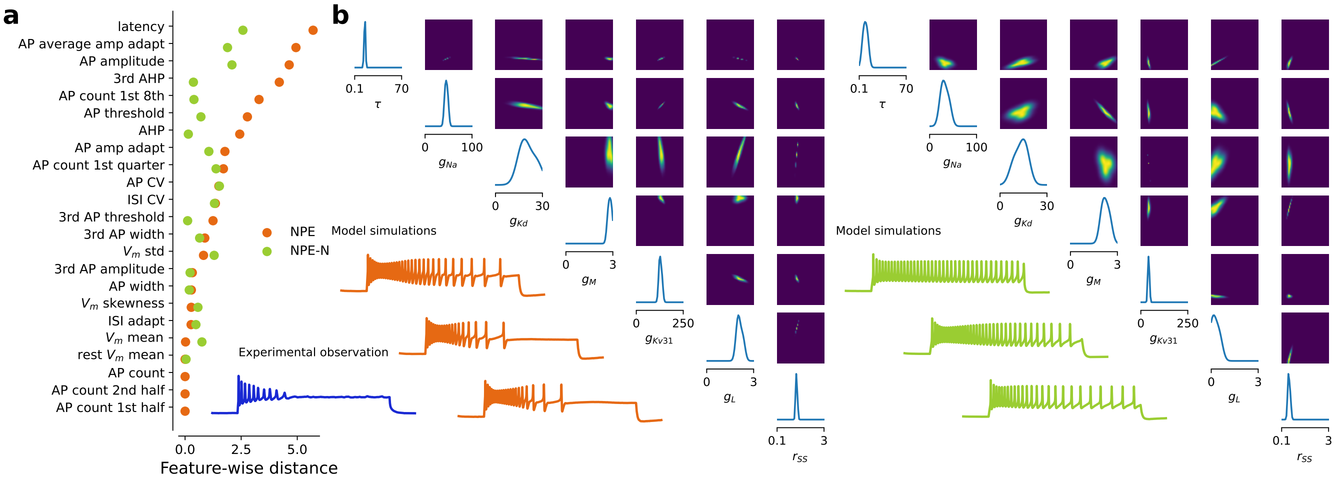

Figure S7 NPE vs NPE-N, illustration 6: ***Sst Th_1*** **interneuron.** Analogous to Fig. 3a,b.

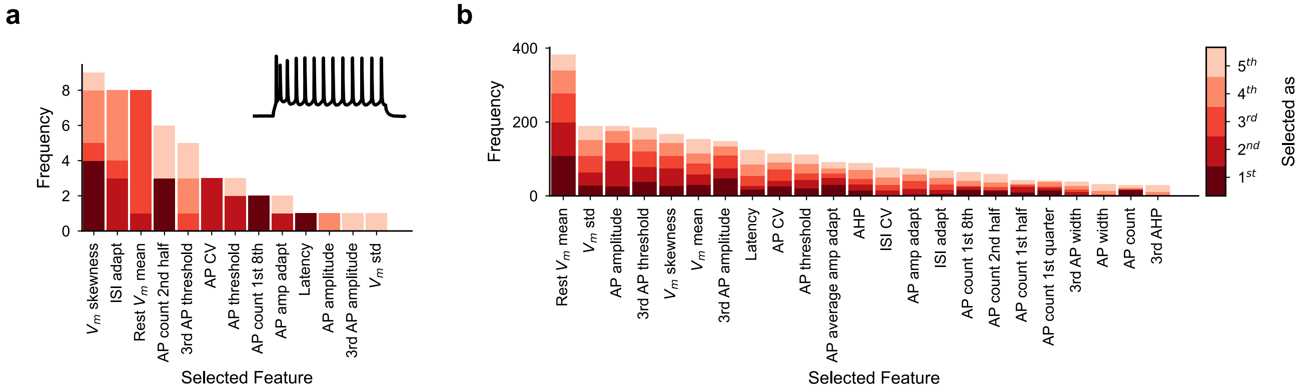

Figure S8 Ranking commonly used electrophysiological features by their ability to constrain posterior estimates. **a** Features are ranked by how often they are strongly constraining the posterior of a Pvalb neuron. Strongly constraining features minimize the KL divergence between posterior estimates subject to all 23 features and estimates considering only five. Important features were selected across 10 repeated runs. Shading indicates the order in which they are selected as part of the top five. Features are ranked in descending order. **b** Summary across all 955 MOp neurons, of which features are strongly constraining the posterior estimates.
